# Real-world performance of HIV low viral load values in diagnosing acute HIV infection in a tertiary care hospital in Beijing, China

**DOI:** 10.1186/s12879-024-09486-8

**Published:** 2024-06-15

**Authors:** Li Li, Xia Feng, Fei Zhao, Defu Yuan, Xizhao An, Xiaoxue Tian, Hao Wu, Bin Su, Tong Zhang, Lifeng Liu

**Affiliations:** 1 Beijing Key Laboratory for HIV/AIDS Research, Sino-French Joint Laboratory for HIV/AIDS Research, Clinical and Research Center for Infectious Diseases, Beijing Youan Hospital, Capital Medical University, Beijing, 100069 China; 2 Department of Clinical Laboratory, Beijing Youan Hospital, Capital Medical University, Beijing, 100069 China; 3 Beijing Youan Hospital, Beijing Key Laboratory for HIV/AIDS Research, Sino-French Joint Laboratory for Research on Humoral Immune Response to HIV Infection, Clinical and Research Center for Infectious Diseases, Capital Medical University, Beijing, 100069 China

**Keywords:** HIV, Low, Viral load values, Diagnosis, Acute HIV infection

## Abstract

**Background:**

Early diagnosis of HIV infection decreases the time from HIV diagnosis to viral suppression and reduces further HIV transmission. The Chinese Guidelines for the Diagnosis and Treatment of HIV/AIDS (2021 edition) state that an HIV RNA level > 5,000 copies/mL is the threshold for diagnosing HIV infection. The impact of low viral load values on HIV diagnosis needs to be investigated.

**Methods:**

There were 3455 human immunodeficiency virus (HIV1 + 2) antibody results (immunoblotting method) and 65,129 HIV viral load values at Beijing Youan Hospital from 2019 to 2022. A total of 2434 patients had both antibody confirmatory results and viral load results. The confirmatory antibody results and HIV viral load results of 2434 patients were analyzed to investigate the impact of low viral load values on HIV diagnosis.

**Results:**

Of the 2434 patients who had both confirmatory antibody results and viral load results, the viral load values of 140 patients (5.8%) had viral loads ranging from 40 copies/mL to 5,000 copies/mL before positive confirmatory antibody result, and of these 140 patients, the sample receipt time for the viral load tests of 96 (66.7%) individuals was 1 to 6 days earlier than the corresponding sample receipt time for the confirmatory antibody test. In addition, 34 patients (1.4%) had low viral loads ranging from 40 copies/mL to 1,000 copies/mL before positive confirmatory antibody result.

**Conclusion:**

This study revealed that there is a risk of missed diagnosis if a threshold of 5000 copies/mL is used for the diagnosis of HIV infection. These data provide valuable information for the early diagnosis of HIV infection, and our findings have potential benefits for decreasing HIV transmission.

## Background

There were 39 million HIV-infected patients and 1.3 million new cases in the world in 2022 [1], and there were 1.053 million people living with HIV at the end of 2020 in China; therefore, HIV/AIDS is still a major public health problem [2]. HIV-1 RNA is detected in plasma approximately 7 days before a HIV p24 antigen test, and 12 days before a HIV specific antibody test [3]. The rate of sexual transmission during acute infection is 3.2 times greater than that during chronic infection [4]. Immediate treatment of acute HIV-1 infection maintains cell count and functionality, limits the size of the viral reservoir, and reduces subsequent viral transmission [5]. Therefore, early diagnosis and treatment of acute HIV-1 infection has been one of the strategies used to reduce disease damage and subsequent viral transmission.

According to the Chinese Guidelines for the Diagnosis and Treatment of HIV/AIDS (2021 edition), for adults, adolescents, and children over 18 months old, a person who meets one of the following criteria can be diagnosed with HIV infection: (1) a positive HIV antibody screening test and positive HIV supplementation test, including either a positive antibody supplementation test, or a positive qualitative nucleic acid test, or a quantitative nucleic acid quantification of more than 5,000 copies/mL; (2) an epidemiology history or AIDS-related clinical manifestations, and positivity for two HIV nucleic acid tests; and (3) a positive HIV isolation test [6]. In the study, we analyzed the confirmatory antibody results and HIV viral load results of 2434 patients to investigate the impact of low viral load values on HIV diagnosis.

## Materials and methods

### Patients and specimen

There were 3455 human immunodeficiency virus (HIV1 + 2) antibody results (immunoblotting method) and 65,129 HIV viral load values in Beijing Youan Hospital from 2019 to 2022, and each sample had its own receipt time. There were 2434 patients with both confirmatory antibody results and viral load results.

The route of infection, antiviral treatment status, and other information was recorded in patient’s infectious disease report card and case follow-up form.

EDTA-anticoagulated whole blood was collected from participants, and fresh plasma specimens were stored at 4 °C and were tested by western blot and The Abbott RealTime HIV‐1 assay within 7 days.

### HIV confirmatory antibody test

HIV antibody testing strategies and procedures were carried out in accordance with the National Technical Specification for HIV Testing (2015 Revision and 2020 Revision). A Western blot assay (HIV Blot 2.2; MP Biomedicals Asia Pacific Pte. Ltd., Singapore) was used to confirm HIV infection, and the assay was performed in accordance with the instructions of the manufacturers. The results were interpreted according to the stringent criteria provided by the manufacturer, and based on the presence/ absence of a selection of bands, the results were classified as negative, indeterminate, or positive.

### HIV viral load assay

The Abbott RealTime HIV-1 assay was used for the quantification of HIV-1 RNA in plasma, and the assay was performed on an automated real‐time PCR‐based m2000 system according to the manufacturers’ instructions. Purified RNA was obtained from 0.6 ml of plasma using an Abbott m2000sp automated extractor, which employs magnetic particle technology, followed by amplification and detection on an Abbott m2000rt real-time PCR instrument. The assay lower limit of quantification (LLQ) is 40 copies/ml, with a dynamic range of 40–10,000,000 copies/ml (0.6 ml input). The target sequence for the Abbott RealTime HIV-1 assay is in the *pol integrase* region of the HIV-1 genome, this region is highly conserved, the assay allows detection of diverse group M subtypes and group O isolates.

### Results analysis

Descriptive statistics, time intervals, and the percentage were calculated using Microsoft 365 Excel.

## Results

### Analysis of the confirmatory antibody results at Beijing Youan Hospital from 2019 to 2022

There were 3455 human immunodeficiency virus (HIV1 + 2) confirmatory antibody results (immunoblotting method) in Beijing Youan Hospital from 2019 to 2022. There were 2586 (74.8%) positive results, 355 (10.3%) indeterminate results, and 514 (14.9%) negative results.

There were 2434 patients with both confirmatory antibody results and viral load results in Beijing Youan Hospital from 2019 to 2022, and 2768 confirmatory antibody results were obtained for these 2434 patients. There were 2222 (80.3%) positive results, 281(10.1%) indeterminate results, and 265 (9.6%) negative results. A total of 187 patients had two or more confirmatory antibody results, the confirmatory antibody results of 79 patients had changed from indeterminate to positive, and 10 patients had low viral load values less than 5,000 copies/mL before positive confirmatory antibody results.

### Analysis of HIV viral load results in Beijing Youan Hospital from 2019 to 2022

There were 65,129 HIV viral load values in Beijing Youan Hospital from 2019 to 2022, and 6.2% of the values ranged from 40 copies/mL to 5000 copies/mL. There were 12,051 viral load results of 2434 patients who had both confirmatory antibody results and viral load results, and 10.2% of the values ranged from 40 copies/mL to 5000 copies/mL.

### Sample receipt time intervals and viral load distribution of 140 individuals who had viral load values ranging from 40 copies/mL to 5000 copies/mL before positive confirmatory antibody results

There were 2434 patients with both confirmatory antibody results and viral load results at Beijing Youan Hospital from 2019 to 2022. Of these 2434 patients, 140 individuals (5.8%) had the viral load values ranging from 40 copies/mL to 5000 copies/mL before positive confirmatory antibody results, and of 140 individuals, 4 individuals had two viral load values. Of these 2434 patients, 91 individuals (3.7%) had the viral loads ranging from 40 copies/mL to 3000 copies/mL before positive confirmatory antibody results, and 34 individuals (1.4%) had the viral loads ranging from 40 copies/mL to 1000 copies/mL before positive confirmatory antibody results.

Each sample of the 2434 patients who had both confirmatory antibody results and viral load results had a receipt time. There were 140 individuals who had viral load values ranging from 40 copies/mL to 5000 copies/mL before positive confirmatory antibody results. In addition, of these 140 individuals, 4 individuals had two viral load values. These 140 individuals had not started treatment with ARVs and had not received some medication that affected their plasma viral load before positive confirmatory antibody results.

**Fig. 1:**
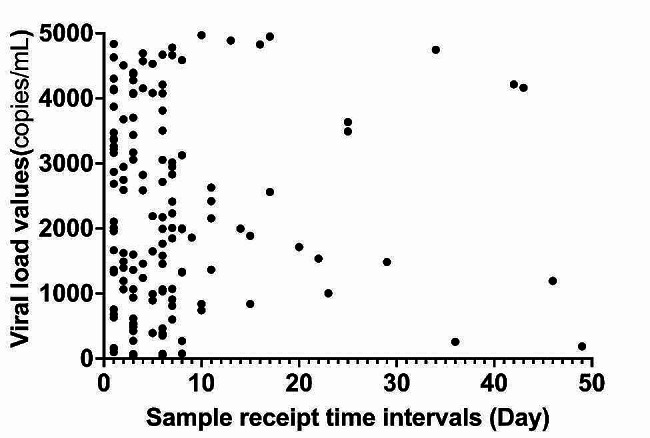
Sample receipt time intervals between the viral load test and the confirmatory antibody test and viral load distribution of 140 individuals who had viral loads ranging from 40 copies/mL to 5000 copies/mL before positive confirmatory antibody results

Sample receipt time intervals between the viral load test and the confirmatory antibody test for 140 individuals who had viral loads ranging from 40 copies/mL to 5000 copies/mL before positive confirmatory antibody results are shown in Fig. 1. Of these 140 individuals, the sample receipt time for the viral load test of 6 individuals was more than 30 days earlier than the corresponding sample receipt time for the confirmatory antibody test, and the sample receipt time for the viral load test of 96 (66.7%) individuals was 1 to 6 days earlier than the corresponding sample receipt time for the confirmatory antibody test. The sample receipt time for the viral load test of 41 individuals was 7 to 29 days earlier than the corresponding sample receipt time for the confirmatory antibody test, in addition, one individual had two viral load values.

The viral load distributions of the 140 individuals who had viral load values ranging from 40 copies/mL to 5000 copies/mL before positive confirmatory antibody results are shown in Fig. 1: 40 to 1000 copies/mL, 34 individuals; 1000 to 2000 copies/mL, 35 individuals; 2000 to 3000 copies/mL, 22 individuals; 3000 to 4000 copies/mL, 19 individuals; and 4000 to 5000 copies/mL, 30 individuals. In addition, three individuals had two values ranging from 1000 to 2000 copies/mL, and one individual had two values ranging from 400 to 1000 copies/mL before positive confirmatory antibody results.

## Discussion

In this study, we analyzed the viral load and confirmatory antibody results at Beijing Youan Hospital from 2019 to 2022. We found that of 2434 patients had both confirmatory antibody results and viral load results, 140 individuals (5.8%) had viral load values ranging from 40 copies/mL to 5000 copies/mL before positive confirmatory antibody results, and of these 140 patients, the sample receipt time for the viral load test of 96(66.7%) individuals was 1 to 6 days earlier than the corresponding sample receipt time for the confirmatory antibody test. In addition, the viral loads of 34 patients (1.4%) ranged from 40 copies/mL to 1,000 copies/mL before positive confirmatory antibody results were obtained.

Abbott RealTime HIV-1 Assay are recommended for HIV-1 RNA monitoring and are not intended for diagnosis of infection. HIV-1 RNA qualitative assays are recommended for diagnosis of infection, but they are not used in the clinical setting in China. HIV-1 RNA quantification diagnostics, such as Abbott RealTime HIV-1 Assay, are being widely used in clinical setting in China. Society of Infectious Diseases Chinese Medical Association and Chinese Center for Disease Control and Prevention has established guidelines for the use of existing quantitative HIV-1 RNA diagnostics for the diagnosis of HIV-1 infection, the proposed threshold is 5,000 copies/mL. According to the US Guidelines for the Use of Antiretroviral Agents in Adults and Adolescents with HIV, in the presence of compatible symptoms or exposure history, a low HIV RNA concentration is consistent with acute HIV infection, the previously proposed threshold is 3,000 copies/ mL [7]. According to the 2021 European guideline on HIV testing in genito-urinary medicine settings, low HIV RNA concentrations (< 1,000 copies/mL) should be interpreted with caution, and HIV infection should be confirmed by providing evidence of ongoing seroconversion in a follow-up specimen 1–2 weeks later [8]. The incorporation of nucleic acid amplification testing into HIV testing is beneficial for diagnosing acute HIV infection and decreasing HIV transmission [9]. In this study, 140 individuals (5.8%) had viral loads raging from 40 copies/mL to 5000 copies/mL before positive confirmatory antibody results, which indicated a risk of missed diagnosis if a threshold of 5000 copies/mL was used for the diagnosis of HIV infection in China. Our findings provide valuable information for the early diagnosis of HIV infection.

HIV RNA and the p24 antigen can be detected approximately 11 to 12 days and 14 to 15 days after infection respectively, and HIV specific antibodies can be detected three to eight weeks after infection [10]. Because HIV specific antibodies appear later than HIV RNA in the blood after infection, one or more HIV viral load tests were done before a positive confirmatory antibody result for high-risk groups. In this study, the viral load values ranging from 40 copies/mL to 5,000 copies/mL of 96 (66.7%) individuals were detected 1 to 6 days earlier than a positive confirmatory antibody result. To diagnose acute HIV-1 infection earlier, more HIV viral load tests should be considered before a positive confirmatory antibody result for the time interval between HIV RNA and HIV specific antibodies appearance. Of the 795 outpatients with HIV-seronegative/HIV-serodiscordant results, 30 patients (3.8%) were diagnosed with acute or early HIV infection after undergoing diagnostic testing with the quantitative Xpert HIV-1 viral load assay [11]. Compared with the fourth-generation HIV antigen/antibody test, the number of acute infected patients increased from 81 to 112 after the addition of pooled nucleic acid testing [12]. A total of 467 HIV antibody-negative samples were tested by HIV pooled nucleic acid testing (Roche Molecular Systems), four (0.9%) of which were HIV-1 RNA positive [13]. In the present study, individuals with low viral loads were identified before positive antibody confirmatory results were obtained.

To investigate the impact of low HIV viral load on HIV diagnosis, contamination during HIV viral load testing must be eliminated. The increased automation of HIV viral load testing reduces cross-contamination and biosafety risks. Pilcher et al. reported two false positive results from nucleic acid amplification tests, these two patients had positive nucleic acid test results at initial testing, but negative results from both nucleic acid amplification and antibody testing at follow-up [14]. As a supplementary HIV testing strategy, the sensitivity and specificity of the reagent should be improved to avoid or reduce the occurrence of false-positive results [15]. Following CAR-T cell therapy, several false positive HIV nucleic acid tests have been reported, and there are cross reactions between lentiviral vectors and HIV genomes targeted in the HIV viral load assays; these false positive results may be related to the use of lentiviral vector [16, 17]. Low HIV RNA concentrations (e.g., < 1,000 copies/mL) may represent false positive results and should be interpreted with caution. False positive results can be excluded by retesting with different reagents, ongoing seroconversion at follow-up, and comprehensive analysis.

In this study, a retrospective analysis was conducted of patients who underwent HIV confirmatory antibody tests and HIV viral load tests at Beijing Youan Hospital from 2019 to 2022. This was a cross-sectional study, and only a few patients had two or more follow-up results. To investigate the impact of low HIV viral load on HIV diagnosis, it is better to establish a prospective cohort and conduct routine follow-up of enrolled patients [18].

In conclusion, the confirmatory antibody results and viral load results of 2434 patients were analyzed to investigate the impact of low viral load values on HIV diagnosis, and 140 patients (5.8%) had viral load values ranging from 40 copies/mL to 5,000 copies/mL before positive confirmatory antibody results. These data provide valuable information for the early diagnosis of HIV infection, and our findings have potential benefits for decreasing HIV transmission.

## Data Availability

The datasets used and analyzed during the current study are available from the corresponding author upon reasonable request.
